# Molecular Origins of Chiral Amplification on an Achiral Surface: 2D Monolayers of Aspartic Acid on Cu(111)

**DOI:** 10.1021/acsnano.2c12312

**Published:** 2023-03-06

**Authors:** Laura A. Cramer, Amanda Larson, Avery S. Daniels, E. Charles H. Sykes, Andrew J. Gellman

**Affiliations:** † Department of Chemistry, Tufts University, Medford, Massachusetts 02155-5813, United States; ^‡^Department of Chemical Engineering and ^§^W.E. Scott Institute for Energy Innovation, Carnegie Mellon University, Pittsburgh, Pennsylvania 15213, United States

**Keywords:** chiral, surface, enantiomers, aspartic acid, Cu(111), scanning tunneling microscopy

## Abstract

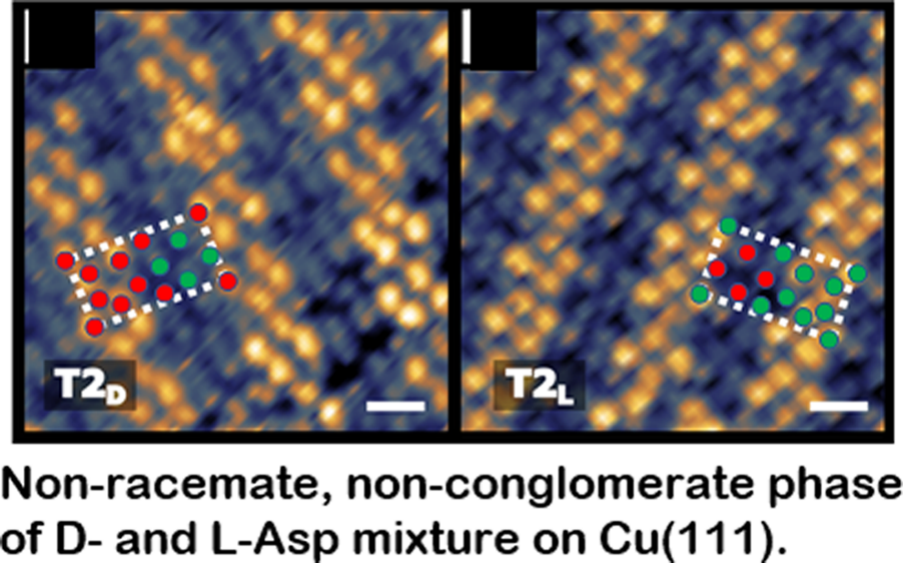

Recent experiments have demonstrated an intriguing phenomenon in which adsorption of a nonracemic mixture of aspartic acid (Asp) enantiomers onto an achiral Cu(111) metal surface leads to autoamplification of surface enantiomeric excess, *ee*_s_, to values well above those of the impinging gas mixtures, *ee*_g_. This is particularly interesting because it demonstrates that a slightly nonracemic mixture of enantiomers can be further purified simply by adsorption onto an achiral surface. In this work, we seek a deeper understanding of this phenomena and apply scanning tunneling microscopy to image the overlayer structures formed by mixed monolayers of d- and l-Asp on Cu(111) over the full range of surface enantiomeric excess; *ee*_s_ = −1 (pure l-Asp) through *ee*_s_ = 0 (racemic dl-Asp) to *ee*_s_ = 1 (pure d-Asp). Both enantiomers of three chiral monolayer structures are observed. One is a conglomerate (enantiomerically pure), another is a racemate (equimolar mixture of d- and l-Asp); however, the third structure accommodates both enantiomers in a 2:1 ratio. Such solid phases of enantiomer mixtures with nonracemic composition are rare in 3D crystals of enantiomers. We argue that, in 2D, the formation of chiral defects in a lattice of one enantiomer is easier than in 3D, simply because the stress associated with the chiral defect in a 2D monolayer of the opposite enantiomer can be dissipated by strain into the space above the surface.

Study of the surface chemistry of chiral adsorbates has been motivated by interest in issues surrounding the origins of homochiral life on Earth^1−4^ and interest in the enantioselective surface processes that underpin the production of enantiomerically pure compounds for use as pharmaceuticals.^5−9^ It has been suggested that surfaces played a role in prebiotic chemistry by serving as substrates for the concentration of otherwise dilute species from the primordial soup. It has also been noted that some of the minerals present at the time were chiral and that this may have played some role in inducing chirality in molecular species adsorbed on their surfaces.^4,10,11^ In this regard, it is worth noting that simple adsorption, even onto an achiral surface, is sufficient to induce chirality into prochiral molecules.^12^ The technological relevance of such chiral surface chemistry arises from the potential to use intrinsically chiral surfaces and achiral surfaces modified by chiral adsorbates to induce enantioselectivity in interfacial processes such as catalysis, separations, and crystal nucleation.^13−15^

The vast majority of studies of the surface chemistry of chiral compounds have focused on the use of enantiomerically pure compounds, racemic (equimolar) mixtures of enantiomers, or prochiral compounds that are rendered chiral by adsorption.^12^ These studies have yielded insights into the transfer of molecular chirality into chirality of translationally ordered molecular domains on surfaces.^16−19^ This is the 2D analogue to understanding the relationship between the structures of chiral molecules and the structure and packing of their 3D crystals. One of the interesting outcomes of this body of work has been the observation that some of the heuristic rules that apply in the 3D case do not seem to apply in 2D. For example, in 3D, racemic mixtures tend to crystallize into conglomerates (physical mixtures of enantiomerically pure crystals) or into racemates (crystals in which each unit cell contains equimolar numbers of both enantiomers). In 3D, racemates are more common than conglomerates but that is not so in 2D.^7,12,20,21^ Furthermore, in 3D it is very rare to find crystals containing both enantiomers of chiral compounds in nonracemic ratios or even as random solid solutions of equimolar mixtures. It is also well-established that in 3D the majority (∼90%) of chiral compounds crystallize from racemic mixtures into racemates, thereby making their purification by crystallization somewhat challenging.^21^ On surfaces, there are frequent observations that deviate from the trends observed in 3D. This presents intriguing opportunities for use of surfaces and interfaces to perform enantioselective reactions and separations.

A recent review of the scanning tunneling microscope (STM) literature has focused on the ordered domains formed by the adsorption of racemic mixtures (and prochiral species that form racemic mixtures of chiral adsorbates) on single crystal metal surfaces.^12^ Analysis of these studies to determine whether the adsorbed mixtures form domains that are enantiomerically pure (homochiral) or domains that are racemic (heterochiral) suggests that these two outcomes are equally likely on surfaces. In other words, chiral molecular adsorption and 2D crystallization does not show the propensity for racemate formation that is found in 3D crystallization.^21^

There are also examples in the surface science literature in which ordered 2D domains containing both enantiomers of a chiral adsorbate exhibit disorder in the sense that enantiomers are randomly distributed on the 2D lattice. This is analogous to the formation of a random solid solution in an alloy. As an example, when a racemic mixture of dl-proline (Pro) is adsorbed on Cu(110), it forms an ordered overlayer in which the d- and l-enantiomers are randomly distributed rather than forming unit cells with equal numbers of each enantiomer.^22−24^ The origin of this behavior can be understood by recognizing that the adsorption of a Pro molecule onto the Cu(110) surface results in the formation of a diastereomer with two chiral centers, one defined by the intrinsic chirality of the Pro and the other by its adsorption footprint on the surface. The footprint dominates in dictating the structure of the adsorbed Pro monolayer such that the footprints form a 2D racemate while the molecular chirality is randomly distributed on the 2D lattice. The ability of systems to accommodate random distributions of chiral centers is rare in 3D crystals of chiral compounds. This behavior in adsorbed monolayers may simply be a result of the ability of the 2D monolayer to relieve stress by relaxation into the third dimension normal to the surface.

A recent study by Ernst et al. has identified a 2D system that forms an ordered structure with unequal numbers of enantiomers in each unit cell.^25^ Trispentahelicene has a structure mimicking a three bladed propeller and exists in enantiomeric forms labeled M- and P-. The STM study of a racemic mixture of trispentahelicene propellers adsorbed on Au(111) revealed that the molecules lie roughly parallel to the Au(111) surface and that the absolute chirality of each molecule is discernible from the STM images. More importantly, coexisting ordered domains with 2:1 or 1:2 ratios of M- and P-enantiomers were observed. As already noted, this behavior is extremely rare in 3D crystallization of racemic mixtures. Note also that starting with a 2:1 mixture of trispentahelicene enantiomers on the surface does not necessarily lead to a monolayer composed only of the 2:1 structure observed during adsorption of a racemic mixture.

As mentioned earlier, the vast majority of studies of chiral adsorbates on surfaces have been conducted using either enantiomerically pure compounds or racemic mixtures as starting points. Needless to say, processes such as enantioselective catalysis or enantioselective adsorption on surfaces involve the exposure of surfaces to a variety of nonracemic mixtures that may behave in a manner that cannot be anticipated solely on the basis of the pure enantiomers and racemic mixtures behaviors. The example that serves as the focus of this work is the equilibrium coadsorption of d- and l-aspartic acid (Asp, HO_2_CCH(NH_2_)CH_2_CO_2_H) on the Cu(111) surface.^26−28^Figure 1 plots the equilibrium adsorption isotherms for three systems exhibiting different behaviors: d-/l-alanine on Cu(3,1,17)^R&S^,^29^d-/l-Asp on Cu(3,1,17)^R&S^,^30^ and d-/l-Asp on Cu(111).^27^ Note that, in our notation, d-/l- refers to enantiomer mixtures of arbitrary composition, distinct from dl- that commonly refers to a racemic mixture. Note also that the Cu(3,1,17) surface is intrinsically chiral^9,31^ whereas the Cu(111) surface is achiral. The isotherms are plotted in the form of surface enantiomeric excess, *ee*_s_ = (θ_D_ – θ_L_)/(θ_D_ + θ_L_), versus gas phase enantiomeric excess, *ee*_g_ = (*P*_D_ – *P*_L_)/(*P*_D_ + *P*_L_), and obtained under conditions in which the surface is saturated with adsorbed amino acid, *i*.*e*., θ_D_ + θ_L_ = 1. With these definitions, pure d- has *ee* = 1, pure l- has *ee* = −1, and the racemic mixture has *ee* = 0. The case of d-/l-alanine on Cu(3,1,17)^R&S^ exhibits nonenantiospecific adsorption, *ee*_s_ = *ee*_g_, in spite of the fact that, at some level, the chiral adsorbates must have an enantiospecific interaction with the chiral surfaces. The case of d-/l-Asp on Cu(3,1,17)^R&S^ clearly reveals enantiospecific interactions between the adsorbate and the chiral surfaces; *i*.*e*., *ee*_s_ ≠ 0 when *ee*_g_ = 0. Most interestingly, the case of d-/l-Asp on Cu(111) exhibits autoamplification of enantiomeric excess on the surface, relative to that in the gas phase, |*ee*_s_| > |*ee*_g_|, in spite of the fact that the surface is achiral. This autoamplification of *ee*_s_ can result from enantiospecific interactions between adsorbed Asp enantiomers, in particular, attractive homochiral interactions.^29^ Recently, we have demonstrated that this behavior is well-described by the 2D Ising model.^26,28^

**Figure 1:**
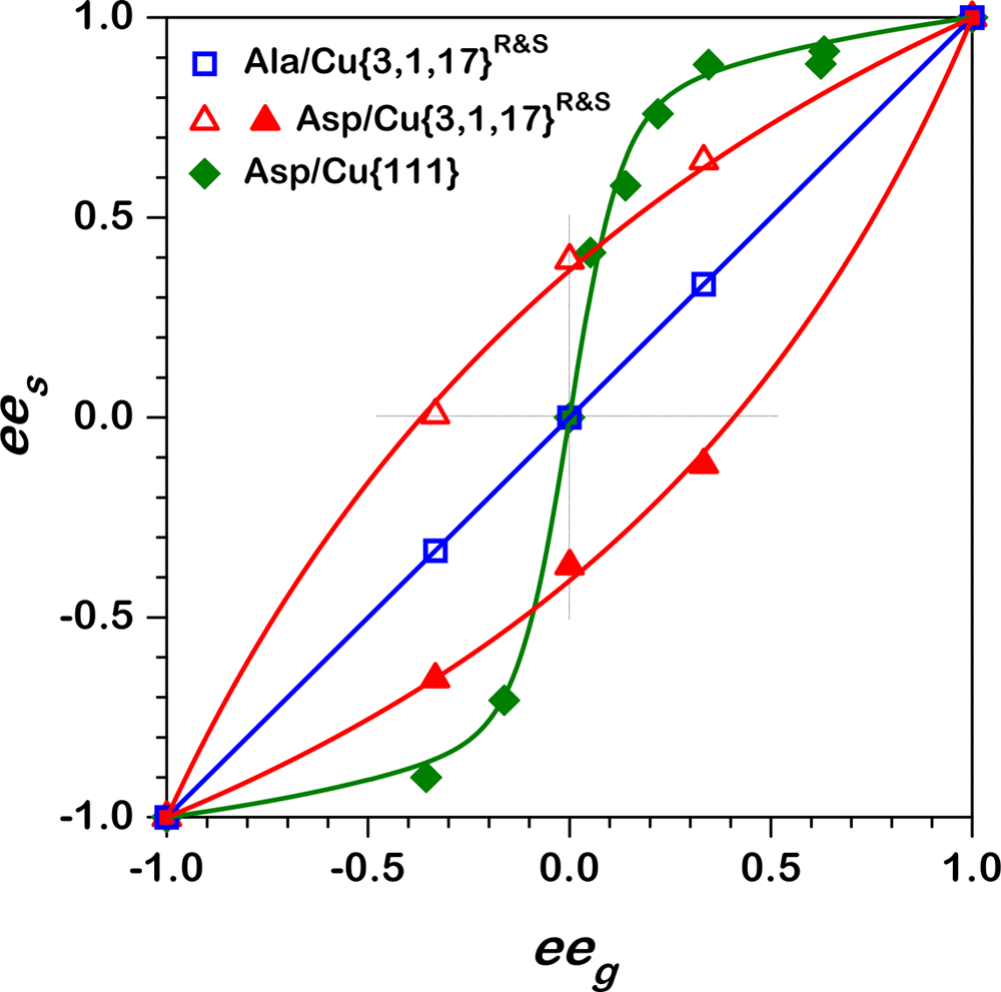
Enantiomeric excess on the surface, *ee*_s_, versus enantiomeric excess in the gas phase, *ee*_g_, for equilibrium adsorption of amino acid mixtures on several Cu(*hkl*) surfaces at 460 K. Alanine (Ala) on Cu(3,1,17) (blue line, open squares) exhibits the results expected for a Langmuir adsorption isotherm with noninteracting adsorbates and no enantiospecific interactions with the surface, *ee*_s_ = *ee*_g_.^29^ Asp on Cu(3,1,17)^R&S^ (red curves, triangles) exhibits Langmuir adsorption with noninteracting adsorbates having enantiospecific interactions with the surface, *ee*_s_ ≠ 0 at *ee*_g_ = 0.^30^ Asp adsorption on achiral Cu(111) (green curve, diamonds) reveals evidence of homochiral intermolecular attractions leading to |*ee*_s_| > |*ee*_g_|.^26,27^

The behavior revealed in Figure 1 motivates the need to study enantiomer adsorption on surfaces under conditions that span the full range of *ee*_s_ ∈ [−1,1]. If the autoamplification of *ee*_s_ arises from intermolecular attractions, this could manifest itself in the structures and compositions of the adsorbed layers formed by d-/l-Asp on Cu(111). Somehow, they must accommodate the continuous nonlinear behavior of *ee*_s_, perhaps by accommodation of surface phases that can adopt compositions other than those of enantiomerically pure conglomerates or achiral racemates. Herein, we use molecular-resolution STM imaging to explore the influence of varying enantiomeric excess on monolayer structure. In this case, we allow *ee*_s_ to vary over the range [−1, 1]. We observe two enantiomeric chiral domain structures associated with the enantiomerically pure monolayers, two enantiomeric domains associated with a racemate phase, and two enantiomeric domains of a third monolayer phase with a composition that has inequivalent populations of the two Asp enantiomers.

## Results/Discussion

The chemistry of Asp on several Cu(*hkl*) single crystal surfaces has been determined through several prior studies using l-Asp isotopomers.^32−35^ Adsorption at *T* < 250 K results in the formation of multilayer films. N 1s X-ray photoemission spectroscopy (XPS) reveals that the multilayer exists in the form of zwitterions with the α-carboxylic acid deprotonated, −CO_2_^–^, and the amine in its anionic state, −NH_3_^+^.^32,33^ Annealing to *T* > 400 K results in the desorption of the multilayer and leaves a saturated monolayer on the surfaces. The same state can be achieved by Asp adsorption at a surface temperature *T* > 400 K. N 1s and O 1s XPS reveal that the monolayer Asp is in the form of a biaspartate species, −CO_2_CH(NH_2_)CH_2_CO_2_—. During heating, the adsorbed Asp decomposes at ∼500 K to yield 2CO_2_, H_2_, and CH_3_CC≡N stoichiometrically.^35^ Use of the 1-^13^C_1_-l-Asp isotopomer reveals that the decomposition is initiated by cleavage of the 3C–4C bond to yield ^12^CO_2_ desorption followed by rapid decomposition of the remaining species, −CO_2_CH(NH_2_)CH_2_−, yielding desorption of ^13^CO_2_, H_2_, and CH_3_CC≡N.

### Ordered Structures of d-/l-Asp Mixtures on Cu(111)

**Figure 2:**
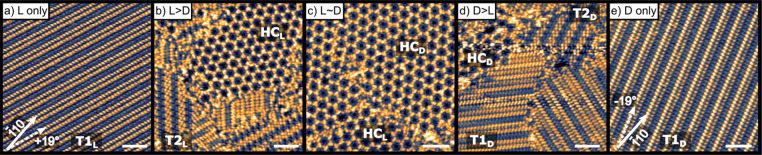
2D Molecular domains formed by d- and l-Asp monolayers of varying *ee*_s_ deposited at 450 K on Cu(111). STM images of the resulting monolayers reveal a variety of domain structures as the *ee*_s_ varies from pure l-Asp to a racemic dl- mixture to pure d-Asp. Panels (a) and (e) reveal the striated chiral domains of pure d- and l-Asp (labeled T1_D_ and T1_L_) that are rotated by ±19° from the [1̅10] direction of the Cu(111) surface. The 3-fold symmetry of the Cu(111) surface results in three equivalent orientations of each striated domain rotated by 60° from one another. Panels (b) and (d) reveal two additional striated domains (T2_L_ and T2_D_) formed when nonracemic mixtures are adsorbed. The orientations of the T2_D_ and T2_L_ domains are aligned with those of T1_D_ and T1_L_, however, their structures differ. Panel (c) reveals two enantiomorphic domains, HC_D_ and HC_L_, of a honeycomb phase that appears for mixtures of adsorbed d-/l-Asp that are close to racemic. When *ee*_s_ deviates significantly from racemic, the HC phases coexist with T2_D_ and T2_L_. Scale bars are 10 nm.

**Figure 3:**
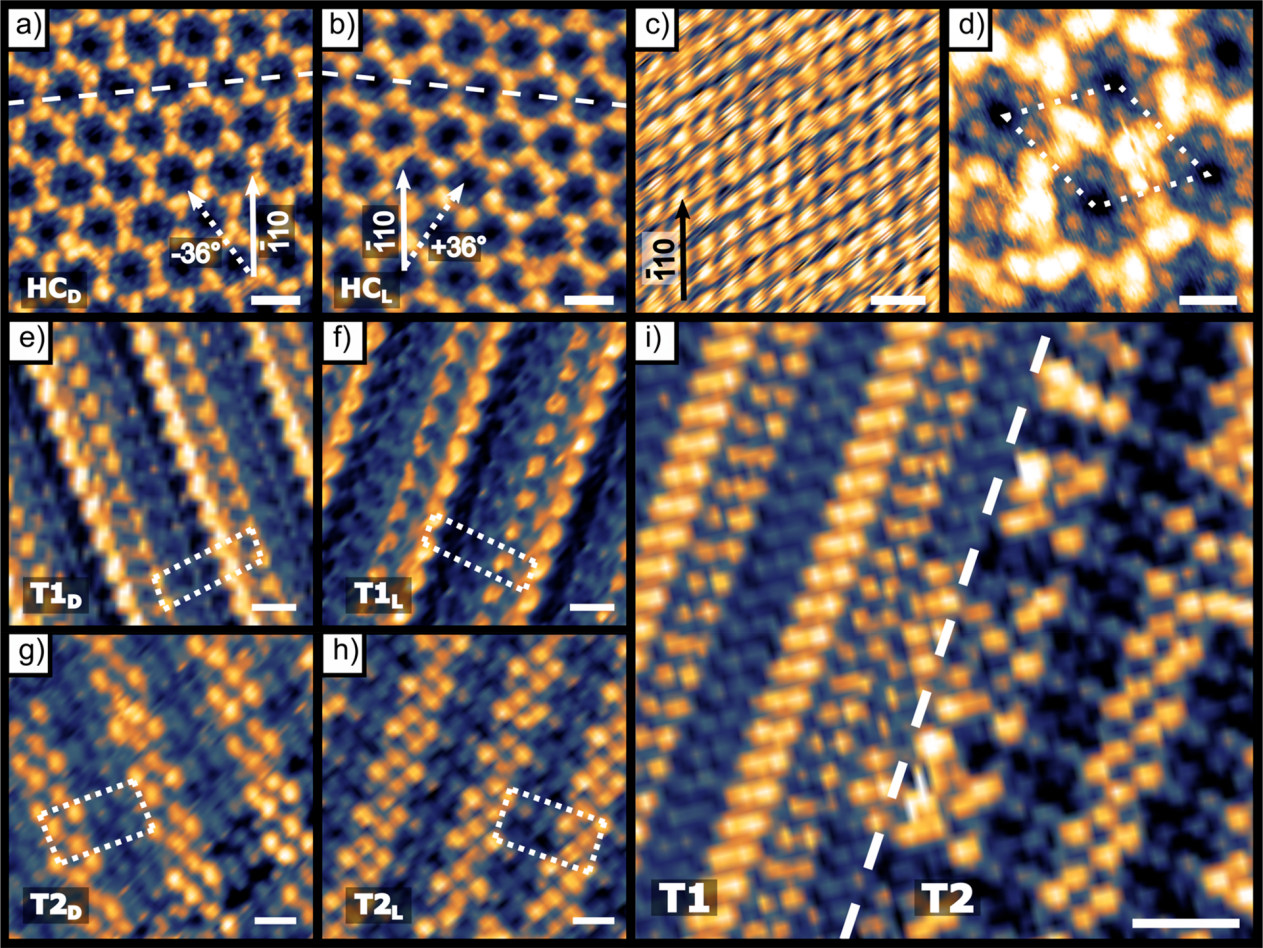
STM images highlighting the mirror symmetry between enantiomorphous domains of d-/l-Asp mixtures on Cu(111). STM images are aligned with the [1̅10] direction of the underlying Cu(111) surface along the vertical direction. Panels (a) and (b) are the two mirror domains of the HC domains (±36° from the [1̅10] direction). Panel (c) is an atomic resolution image of the underlying Cu(111) surface with the [1̅10] direction highlighted (black arrow). Panel (d) is a high-magnification image of one unit cell of the honeycomb structure. Panels (e) and (f) depict the enantiomorphous T1 structure of the pure d-Asp (−19°) domain and the pure l-Asp domain (+19°). Panels (g) and (h) depict the enantiomorphous structures of the T2 domains that form when θ_D-Asp_ > θ_L-Asp_ (−19°) and θ_L-Asp_ > θ_D-Asp_ (+19°). Panel (i) is a high-magnification image showing the boundary between the T1_L_ domain of pure l-Asp and the T2_L_ structure with θ_L-Asp_ > θ_D-Asp_. The boundary between the two domains is highlighted by the white dotted line. Scale bars for (a), (b), and (i) are 2 nm, (c) is 0.5 nm, and (d–h) are 1 nm.

STM images of the monolayer structures formed after deposition of d- and l-Asp with ratios of *ee*_s_ spanning [−1, 1] are shown in Figure 2. At monolayer coverage, enantiomerically pure d- and l-Asp (Figure 2a,e) form chiral striated domains that are rotated ±19° from the close packed [1̅10] direction. These domains are labeled T1_D_ and T1_L_. The rows of T1_L_ (*ee*_s_ = −1) are oriented +19°, while the rows of T1_D_ (*ee*_s_ = 1) are oriented −19° from the [1̅10] directions of the underlying Cu(111) surface. Due to the 3-fold rotational symmetry of the underlying Cu(111) surface, the chiral T1 domains both occur in three equivalent orientations rotated by 120° from one another. Panels b and d of Figure 2 reveal a second type of striated domain, which we have labeled T2. These structures form from enantiomer mixtures when there is an excess of one enantiomer on the surface; hence, they are labeled T2_D_ and T2_L_. As is the case for the enantiopure T_1_ domains, the rows of these T2 domains are rotated by ±19° from the underlying Cu(111) [1̅10] direction. Adsorption of racemic Asp on Cu(111) leads to the formation of a third ordered overlayer named the honeycomb structure, HC. A similar honeycomb structure has been observed for alanine on Cu(111).^36,37^ This dl-Asp HC structure is present in two orientations with respect to the underlying Cu(111) surface, designated HC_D_ and HC_L_. The HC domains are also chiral by virtue of their orientation relative to the underlying Cu(111) surface. HC_D_ domains are oriented −36° from the [1̅10] direction, while HC_L_ domains are oriented +36° from the [1̅10] direction (Figure 3a,b). Because the HC domains have 3-fold rotational symmetry, their rotation by 120° is indistinguishable from the initial structure. The rotational orientation of the HC domains is dictated by the enantiomer in slight excess. This is shown by a correlation of the HC domain rotation with the orientation of the surrounding T1 and T2 domains as shown in Figure 2b,d. For example, when l-Asp is in excess (Figure 2b), a HC_L_ domain can be seen surrounded by T2_L_ domains. The same can be seen in Figure 2d where the presence of T1_D_ is accompanied by HC_D_ domains.

The unit cell matrix assignments of the three ordered overlayer structures are illustrated in Figures SI1–SI3 and discussed in the Supporting Information. Briefly, the master matrices for the enantiomers of the three structures are , , and , consistent with the convention proposed by Ernst et al.^38^ The matrix representations of the ordered overlayers shown in Figures 2 and 3 are given in the Supporting Information Section SI1. The T1, T2, and HC unit cells have areas of *A* = 1.99, 3.84, and 4.42 nm^2^, respectively. Based on features in the STM images and on the unit cell areas, we find that the T1, T2, and HC unit cells contain, 6, 12, and 12 molecules of Asp, respectively, with similar areal densities of ρ_2D_ = 3.02, 3.13, and 2.71 Asp/nm^2^.

Figure 3 shows high-resolution STM images of the monolayer structures observed upon deposition of d-/l-Asp mixtures onto Cu(111). All the images have been rotated so that the [1̅10] direction of the underlying Cu(111) surface is oriented vertically (see Figure 3c for an atomically resolved clean Cu(111) STM image) in order to highlight the mirror symmetry of the l- and d-Asp domains. Panels a, b, and d of Figure 3 show the HC domains formed by the racemic mixture of adsorbed d-/l-Asp. The dashed white lines in Figure 3a,b reveal the mirror symmetry of the two HC enantiomers about the [1̅10] substrate direction. The high-resolution image in Figure 3d reveals six small features arranged in a hexagon inside each honeycomb cell. These features are spaced by ∼0.6 nm. They are surrounded by six groups of raised trimers that are shared between three hexagonal cells at their common vertex. The monomers in these trimers can appear bright or dim and the distribution of the bright and dim features appears to be random. If this is revealing different numbers of enantiomers in these vertex trimers, it may also be revealing the type of structural flexibility that allows for domains that are neither conglomerates nor racemates. Panels e and f of Figure 3 depict the enantiomorphs of the T1 row structures of the pure d-Asp (−19°) and the pure l-Asp (+19°) domains. The T1 unit cells are outlined with dashed white lines and have dimensions of 0.67 nm × 3.03 nm. Panels g and h of Figure 3 depict the enantiomorphous structures of the T2 domains having *ee*_s_ > 0 and a domain orientation angle of −19° when d-Asp is in excess and having *ee*_s_ < 0 and an angle of +19° when l-Asp is in excess. The unit cell of the T2 structure is outlined with a dashed white line. The T2 domains form unit cells of dimensions 1.28 nm × 2.70 nm. Note that this is roughly twice the short dimensionto the T1 unit cell dimensions. Figure 3i shows a high-resolution image revealing a boundary between the T1_L_ domain of pure l-Asp and a T2_L_ domain. The boundary between the two domains is highlighted by the dashed white line.

**Figure 4:**
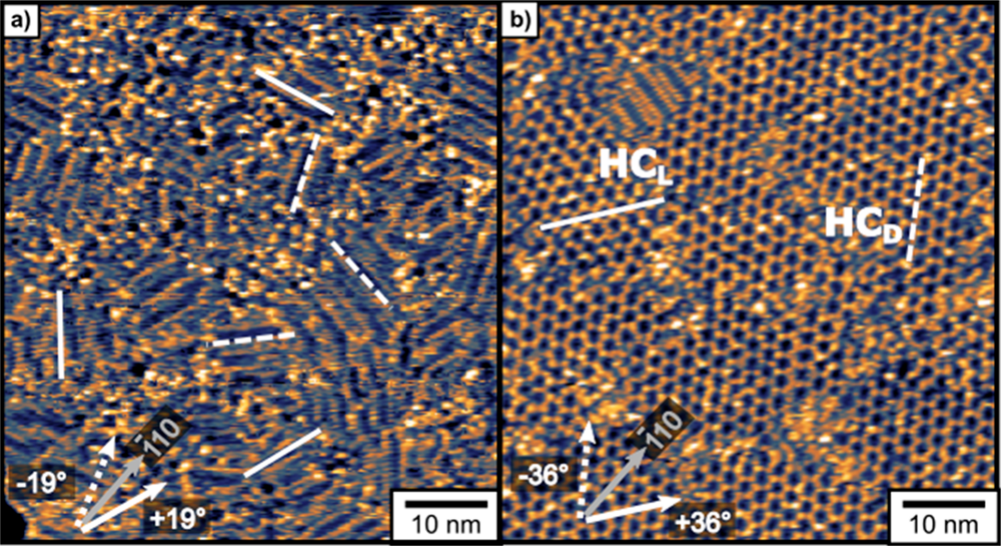
(a) STM image of the Asp monolayer on Cu(111) after codosing a racemic mixture of d- and l-Asp followed by three consecutive doses of l-Asp (30 min at ∼0.04 ML/min and 450 K) followed by three consecutive doses of d-Asp (same conditions). The result is a conglomerate phase with small striated domains interspersed with disordered regions. (b) Annealing the same Asp monolayer for 6 min at 450 K in vacuum yields domains of the racemate HC phase. HC_D_ and HC_L_ domains are indicated by the dashed and solid white lines.

One question to address is the relative thermodynamic stabilities of the conglomerate T1 and racemate HC phases. Figure 4a shows the results of an experiment in which consecutive doses of individual enantiomers were used to generate a roughly racemic, but not equilibrated, surface composition. This experiment began with the codeposition of equimolar d- and l-Asp sufficient to saturate the Cu(111) surface at *T* = 450 K. This resulted in the formation of an overlayer formed of HC domains. This surface was then exposed three times, consecutively, to l-Asp (30 min at ∼0.04 ML/min and 450 K) followed by three consecutive exposures to d-Asp (same conditions). The surface was imaged at room temperature after each exposure. The exposures to l-Asp resulted in displacement of a fraction of the adsorbed d-Asp and the subsequent exposure to d-Asp displaced some of the l-Asp. Upon recovery of a roughly racemic coverage, we observed the formation of randomly distributed T1_D_ and T1_L_ domains (Figure 4a). The solid white lines indicate T1_L_ domains and dashed lines indicate T1_D_ domains. Annealing this semiordered phase in a vacuum at 450 K for 6 min led to its transformation into larger ordered patches of both HC_L_ and HC_D_ structures. This revealed the spontaneous transformation of an overall racemic mix of small enantiopure T1 domains (conglomerate) into the more highly ordered HC phase (racemate) upon annealing. This suggests that the racemate HC phase is thermodynamically stable relative to the conglomerate T1 phase.

**Figure 5:**
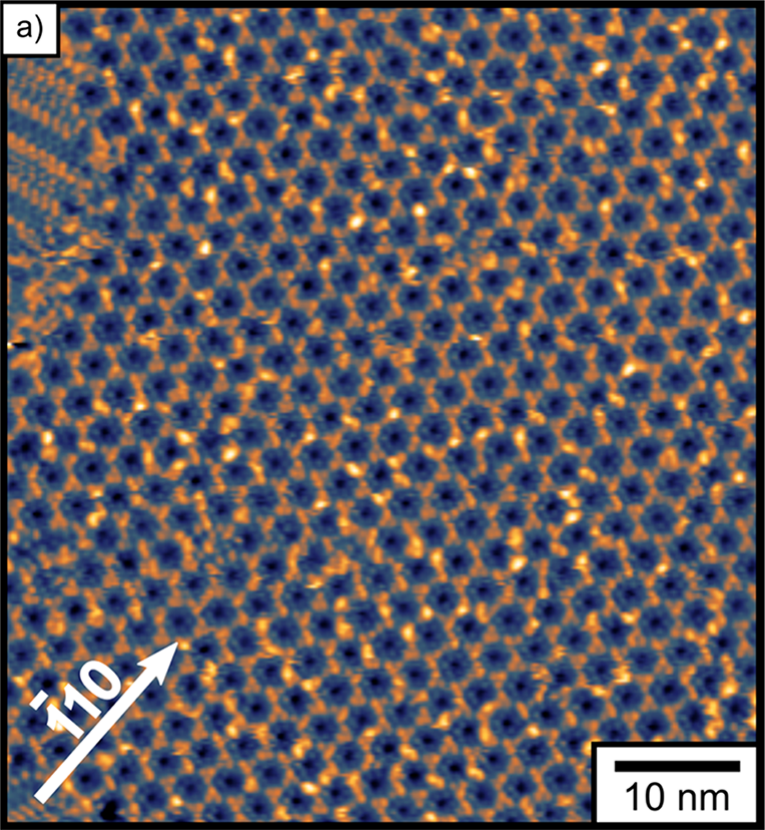
Large area STM image of a single HC domain. Randomly distributed bright spots appear only in the outer walls of the HC unit cells. This suggests that possible strain resulting from incorporation of an enantiomeric excess of one enantiomer into the otherwise racemate HC domain.

Careful examination of high-resolution large-scale STM images of HC domains such as the one in Figure 5 reveals randomly distributed bright spots appearing as protrusions. These features appear along the outer walls of the individual hexagons forming the HC domain. There is no order to these bright protrusions. The appearance of these randomly distributed bright spots is suggestive of a structure that has long-range order but local disorder in terms of individual molecular assembly. It is possible that these are enantiomer defects at which a slight excess of one enantiomer is randomly incorporated into the racemate lattice of the HC domains.

### d-/l-Asp Overlayer Structure and Composition versus Gas Phase Composition

One of the distinguishing features of this work is the collection of STM images of enantiomer mixtures across the full range of surface enantiomeric excess. After adsorption of d-Asp and 1,4-^13^C_2_-l-Asp mixtures on Cu(111), many STM images were collected and then analyzed to determine the fraction of the surface covered by each of the T1, T2, and HC structures and the fraction that was classified as “disordered”. After STM imaging, the surface was heated to decompose the adsorbed Asp. The values of *ee*_s_ were quantified by using mass spectrometry to determine the ratios of CO_2_ and ^13^CO_2_ generated by d-Asp and 1,4-^13^C_2_-l-Asp decomposition, respectively. Table 1 summarizes surface compositions and the fraction of the surface covered by each of the domain types described above.

**Table 1:** Fractional Domain Coverages for Enantiomer Surface Excesses Spanning *ee*_s_ ∈ [−1,1]^a^

*ee*_s_^expt^	*θ*_T1_L__	*θ*_T2_L__	*θ*_HC_L__	*θ*_HC_D__	*θ*_T2_D__	*θ*_T1_D__	*θ*_dis_	*ee*_s_^mdl^	*χ*_*i*_^2^
1.00						1		1.00	0.000
0.85				0.01	0.24	0.45	0.30	0.80	0.003
0.47				0.15	0.04	0.80	0.02	0.82	0.125
0.02			0.03	0.9			0.07	0.00	0.001
–0.07	0.01		0.51	0.32			0.16	–0.02	0.002
–0.41	0.28	0.31	0.21	0.07			0.14	–0.46	0.002
–0.79	0.35	0.2					0.45	–0.78	0.000
–1.00	1							–1.00	0.000
								*χ*^2^ =	**0.008**

a *ee*_s_^expt^- experimentally measured *ee*_s_, *ee*_s_^mdl^- model predicted *ee*_s_, θ_dis_ - fractional surface coverage of disordered phase.

### Enantiomeric Composition of d-/l-Asp Phases on Cu(111)

We have identified three types of chiral domains, (T1, T2, HC) that are formed by the coadsorption of d- and l-Asp at monolayer coverage on the Cu(111) surface. The T1_D_ and T1_L_ domains are associated with enantiomerically pure monolayers of d- and l-Asp, respectively. The HC_D_ and HC_L_ domains occur in monolayers that are close to racemic, with the chirality of the HC domains seemingly dictated by slight enantiomeric excess in the adsorbed Asp. The T2 domains occur when *ee*_s_ ≠ 0, ± 1, suggesting that they have a composition that is neither conglomerate nor racemate. In addition, many of the STM images include regions that are disordered.

We have quantified the composition of the T2 domains based on the data in Table 1. The left-hand column lists the eight values of *ee*_s_^expt^ determined experimentally for the mixed monolayers of d-/l-Asp enantiomers on the Cu(111) surface. Columns 2–8 list the fractional coverages of each phase, θ_ph_, where ph ∈ [T1_D_, T1_L_, T2_D_, T2_L_, HC_D_, HC_L_, dis], as determined from a set of 88 STM images spanning a total area of 4960 nm^2^ with surface compositions spanning *ee*_s_ ∈ [−1,1]. These images were analyzed using the Scanning Probe Image Processor (SPIP) software package. The domain boundaries were identified for each image and the phase of each domain was assigned from ph ∈ [T1_D_, T1_L_, T2_D_, T2_L_, HC_D_, HC_L_, dis]. SPIP then calculated the surface area within each image that is covered by each of the seven phases. For a given value of *ee*_s_^expt^, the fractional coverage of a given phase, θ_ph_(*ee*_s_^expt^), was determined from the total area covered by that phase, *A*_ph_(*ee*_s_^expt^), divided by the total area of the images obtained at that value of *ee*_s_^expt^. The values of θ_ph_(*ee*_s_^expt^) serve as inputs into a model for estimating the values of
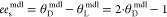
1where
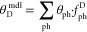
2and *f*_ph_^D^ = 1 – *f*_ph_^L^ is the fractional composition of d-Asp in a given phase. The values of θ_ph_ at each *ee*_s_^expt^ come from the STM measurements listed in Table 1.

The fractional compositions of the T1 and HC phases are constrained at the conglomerate and racemate values, respectively: *f*_T1_D__^D^ = *f*_T1_L__^L^ = 1, *f*_T1_L__^D^ = *f*_T1_D__^L^ = 0, and *f*_HC_L__^D^ = *f*_HC_L__^L^ = *f*_HC_D__^D^ = *f*_HC_D__^L^ = 0.5. In this notation, *f*_T1_D__^D^ denotes the fraction of d-Asp in the T1_D_ phase. Lastly, we have to consider the composition of the disordered regions *f*_dis_^D^ = 1 – *f*_dis_^L^. We have chosen to assume that the disordered regions have compositions that reflect the overall composition of the surface as determined experimentally; *i*.*e*., *f*_dis_^D^ = θ_D_^expt^ = 1/2(*ee*_s_^expt^ + 1).

**Figure 6:**
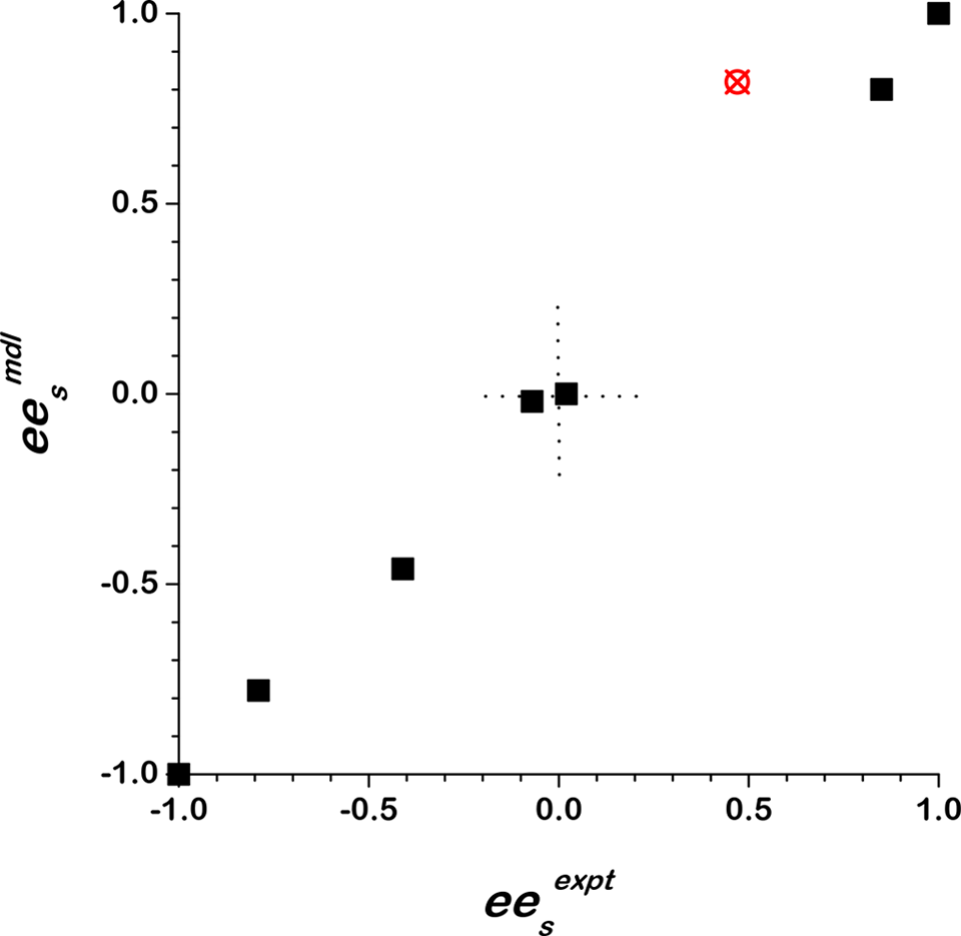
Correlation of *ee*_s_^mdl^ with *ee*_s_^expt^ using *f*_T2_D__^D^ = 0.70, the value yielding the best fit of the model to the data. The outlier point in red at *ee*_s_^expt^ = 0.47 has been excluded from the fit.

Finally, it remains to solve for the only unknown parameter in the model *f*_T2_*D*__^D^ = 1 – *f*_T2_D__^L^ = *f*_T2_L__^*L*^ = 1 – *f*_T2_L__^D^. This is accomplished by minimizing
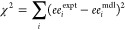
3with respect to *f*_T2_D__^D^ where the summation is over all eight experiments listed in Table 1. This analysis predicts that the T2_D_ phase consists of *f*_T2_D__^D^ = 0.70 d-Asp and *f*_T2_D__^L^ = 0.30 l-Asp or roughly a 2:1 ratio of enantiomers. Figure 6 illustrates the correlation between modeled and measured values of *ee*_s_ for the eight experiments. The correlation is strong, with the exception of the experiment having *ee*_s_^expt^ = 0.47, which has been excluded from the analysis. Inclusion of that data point in the fitting yielded a value of *f*_T2_D__^D^= 0.66, *i*.*e*., it does not change the conclusion regarding the nonracemic composition of the T2_D_ and T2_L_ phases.

An obvious question to address at this point would be the molecular structure of the Asp adsorbed on Cu(111) that leads to the nonracemate composition of the T2_D_ and T2_L_ phases. Based on the unit cell assignments described in the Supporting Information Section SI1, the T1, T2, and HC unit cells contain 6, 12, and 12 Asp species, respectively, with similar areal densities of 3.02, 3.13, and 2.71 Asp/nm^2^. While our STM images can resolve Asp molecules on the surface, they do not have the submolecular resolution needed to identify the orientation of the Asp adsorbate and they certainly do not have the atomic resolution needed to identify their chirality. Using STM to make assignments of absolute chirality for enantiomers of small molecules such as amino acids is extremely rare, especially when imaging enantiomer mixtures.^22,25,39,40^ A key feature of this work has been the development of enantiospecific isotopic labeling coupled with temperature programmed desorption, as a means of quantifying surface enantiomeric excess without the need to count enantiomers in STM images. As such, in the Supporting Information Section SI2 (Figure SI4), we have limited ourselves to providing hypothetical suggestions for the distributions of the Asp enantiomers within the unit cells observed at different surface enantiomeric excesses for Asp on Cu(111).

The implications of the analysis above is that the T2 domain structure observed for Asp enantiomer mixtures adsorbed on the Cu(111) surface is neither a conglomerate nor a racemate. We find that the composition of the T2_D_ domains is roughly 2d- to 1l-Asp and that the composition of the T2_*L*_ domains is 2l- to 1d-Asp. As noted earlier, the formation of phases that are neither racemate nor conglomerate is very rare in 3D crystallization of enantiomer mixtures.^21^ There is some evidence that in 2D this is more common. During d- and l-Pro adsorption on Cu(110), Raval et al. observed the formation of an ordered structure having a random distribution of Pro enantiomers.^22−24^ In that case, chirality of their footprints on the surface formed a racemate structure. More recently, Ernst et al. have used STM to image the structures formed by trispentahelicene enantiomers on Au(111).^25^ In that system, the adsorbates are large enough that their absolute chirality can be determined from the STM images. This reveals ordered 2D domains in which the ratio of enantiomers is 2:1, as observed in this work.

Based on the observations of this work and those just mentioned, it seems that mixed enantiomer domains with nonracemate compositions may be more common in 2D monolayers than in 3D crystals. This can be rationalized by the fact that the vacuum side of the monolayer can accommodate strain associated with enantiomer defects in the adsorbed monolayer, whereas the 3D crystal lattice is much more rigid. Furthermore, our data indicate that the packing structure of adsorbed Asp evolves dynamically in response to differences in *ee*_g_ of the gas phase Asp flux and that surfaces with *ee*_s_ ≠ 1 are composed of coexisting domains of ordered structures (T1, T2, and HC) in ratios that result in an amplification of the gas phase *ee*_g_. Our molecular-scale imaging reveals that, depending on the surface *ee*_s_, some packing structures are energetically preferred. For example, striated domains will spontaneously rearrange into honeycomb domains, if *ee*_s_ ≈ 0. While accurate quantification of the relative energies of the different Asp domains is beyond the ability of current DFT methods due to their large unit cells, associated surface reconstructions, and the energetics of domain boundaries, we hypothesize that the effect of chiral amplification on an achiral surface originates from subtle energetic differences in the preferred packing structures of the Asp enantiomers. For example, as the surface composition drops to *ee*_s_ < 1, T2 domains become preferred over T1 domains and eventually HC domains dominate as the surface approaches *ee*_s_ = 0. Importantly, and unlike 3D crystals of enantiomers, the T2_D_ and T2_L_ domains have composition ratios of θ_D_:θ_L_ ≈ 2:1 and ≈1:2, respectively; *i*.*e*., they are 2D ordered phases that are neither conglomerates nor racemates. It is not clear whether they are enantiomerically ordered structures or random solid solutions of enantiomers. This phenomenon, whereby energetically preferred nonracemic molecular structures on surfaces leads to the amplification of surface *ee*_s_, may be generally true. For example, it has been shown that chromatographic separation columns with achiral stationary media can be used to amplify the *ee* of chiral compounds.^41,42^ While it is impossible to understand those interactions at the molecular level due to the complexity of the interfaces in chromatographic columns, the well-defined nature of the Asp/Cu(111) system has enabled us to relate the *ee*_s_ amplification phenomenon to preferred surface packing structures of the molecules themselves and offer an explanation for their chiral amplification.

### Packing of d- and l-Asp in 2D Phases Observed on Cu(111)

Ideally, one would like to assign features in the STM images of molecular monolayers to structural features of their constituent molecules. In our case, one would like to take this a step further to identify the absolute configurations of individual chiral molecules. This would enable us to make quantitative assessments of the enantiomer compositions of 2D ordered structures formed by coadsorbed enantiomers of chiral molecules. This is all possible for monolayers formed by large and structurally rigid molecules imaged with a tip capable of high resolution.^22,24,25,39^ Unfortunately, Asp is a relatively small molecule that can adopt a number of different configurations through rotations about its C–C bonds. To the best of our knowledge, proline is the only amino acid for which the absolute configuration has been determined directly from STM images.^39^ It is important to note that the ring structure of proline makes it anomalously rigid relative to other amino acids.^22^

In this work, we have circumvented the limitations of STM imaging of adsorbed Asp enantiomers by directly measuring *ee*_s_ using TPRS and mixed monoalayers formed by d-Asp and isotopically labeled 1,4-^13^C_2_-l-Asp. Given the measured *ee*_s_^expt^, we constructed a simple linear model with one degree of freedom, *f*_T2_D__^D^, that can be estimated by finding its value that minimizes the squared difference between the values of *ee*_s_^expt^ and *ee*_s_^mdl^. While we cannot determine the structures or chirality of the adsorbed Asp enantiomers with atomic precision from the STM images in Figure 3, we do gain some insight sufficient to make statements and propose hypothetical suggestions for the enantiomer distribution in the unit cells of the overlayers observed. Figure SI4 shows a subset of the images in Figure 3 with hypothetical suggestions for the unit cell contents superimposed.

## Conclusions

In order to examine the molecular origins of the intriguing phenomenon leading to adsorbed phase *ee*_s_ being amplified relative to the gas phase *ee*_g_ by equilibrium adsorption on achiral surfaces, we performed molecular-scale imaging that examined how the surface packing structures of Asp on Cu(111) evolved as a function of the gas phase *ee*_g_. STM imaging revealed that mixed d- and l-Asp monolayers on the Cu(111) surface with enantiomer compositions ranging from pure d-Asp through racemic dl-Asp to pure l-Asp have six chiral ordered overlayer domains. There are two enantiomerically pure conglomerate domains (T1_D_ and T1_L_), two enantiomorphous racemate domains (HC_D_ and *H*C_L_) and two enantiomorphous domains (T2_D_ and T2_L_). These T2 domains have enantiomer composition ratios of ∼2:1 and ∼1:2 respectively; *i*.*e*., they are ordered phases that are neither conglomerates nor racemates. Our analysis indicates that, depending on the gas phase *ee*_g_, certain surface packing structures with specific ratios of enantiomers are preferred, and we hypothesize that subtle energetic differences between these phases at different values of surface *ee*_s_ drive the chiral amplification effect observed on the achiral Cu(111) surface.

## Methods/Experimental

All STM experiments were performed on a variable-temperature scanning tunneling microscope (Omicron Nanotechnology) in a UHV chamber with a base pressure of <5.0 × 10^–11^ mbar. A Cu(111) single crystal was prepared *via* repeated cycles of Ar^+^ sputtering and annealing to 1000 K in a preparation chamber with a base pressure of <1.0 × 10^–10^ mbar. An Extorr XT100 M residual gas analyzer was used to identify and track gas phase species during Asp deposition onto Cu(111) and during its thermal decomposition. d-Asp and 1,4-^13^C_2_-l-Asp were deposited onto Cu(111) either simultaneously or separately by resistively heating two individual cells in a four-cell Knudsen evaporator. 1,4-^13^C_2_-l-Asp was used in addition to d-Asp to allow for mass spectrometric identification and quantification of the two enantiomers. During deposition, the background pressures of the two enantiomers could be monitored independently because d-Asp has an intense ionization fragment signal at *m*/*z* = 44 while 1,4-^13^C_2_-l-Asp has a corresponding signal at *m*/*z* = 45. The rates of d-Asp and 1,4-^13^C_2_-l-Asp deposition were controlled independently by adjustment of the power going to their respective Knudsen cells. Deposition was carried out at a rate of ∼0.04 ML min^–1^. Unless otherwise stated, the Cu(111) crystal was held at 450 K during all depositions to allow for equilibration of the adsorbed enantiomer composition with the enantiomer composition of the gas phase.^27,30^ STM imaging was performed at room temperature after transfer of the Cu(111) crystal from the preparation chamber to the STM chamber under UHV. After STM imaging, the enantiomer coverage ratio was determined by heating the Cu(111) surface to *T* > 500 K to induce Asp decomposition into CO_2_, CH_3_C≡N, and H_2_. The ratio of adsorbed enantiomers was quantified by monitoring the ratio of the mass spectrometer signals at *m*/*z* = 44 (CO_2_ from d-Asp) and *m*/*z* = 45 (^13^CO_2_ from 1,4-^13^C_2_-l-Asp) integrated over the heating cycle.

## Supporting Information Available

Assignment of the matrix notation and master matrices for the T1_D_, T1_L_, T2_D_, T2_L_, HC_D_, and HC_L_ overlayer phases observed for mixed d-Asp and l-Asp monolayers on Cu(111) using scanning tunneling microscopy and hypothesized enantiomer distributions in the unit cells of the T1_D_, T1_L_, T2_D_, T2_L_, HC_D_, and HC_L_ overlayer phases (PDF)

## References

[ref1] Bada J. L. Origins of homochirality. Nature 1995, 374, 594 10.1038/374594a0. 7536302

[ref2] Bonner W. A. The origin and amplification of biomolecular chirality. Origins of Life and Evolution of the Biosphere 1991, 21 (2), 59–111. 10.1007/BF01809580. 1758688

[ref3] Klussmann M.; Blackmond D. G. Origin of Homochirality. Chemical Evolution Ii: From the Origins of Life to Modern Society 2010, 1025, 133–145. 10.1021/bk-2009-1025.ch007.

[ref4] Hazen R. M.; Filley T. R.; Goodfriend G. A. Selective adsorption of L- and D-amino acids on calcite: Implications for biochemical homochirality. Proc. Natl. Acad. Sci. U. S. A. 2001, 98 (10), 5487–5490. 10.1073/pnas.101085998. 11331767 PMC33239

[ref5] Nguyen L. A.; He H.; Pham-Huy C. Chiral drugs: an overview. Int. J. Biomed. Sci. 2006, 2 (2), 85–100. 23674971 PMC3614593

[ref6] Raval R. Assembling molecular guidance systems for heterogeneous enantioselective catalysis. Cattech 2001, 5 (1), 12–28. 10.1023/A:1011975701856.

[ref7] Shukla N.; Gellman A. J. Chiral metal surfaces for enantioselective processes. Nat. Mater. 2020, 19 (9), 939–945. 10.1038/s41563-020-0734-4. 32747699

[ref8] Stinson S. C. Chiral pharmaceuticals. Chem. Eng. News 2001, 79 (40), 79–97. 10.1021/cen-v079n040.p079.

[ref9] Gellman A. J. An Account of Chiral Metal Surfaces and Their Enantiospecific Chemistry. Accounts Mater. Res. 2021, 2 (11), 1024–1032. 10.1021/accountsmr.1c00145.

[ref10] Bonner W. A.; Kavasmaneck P. R.; Martin F. S.; Flores J. J. Asymmetric Adsorption of Alanine by Quartz. Science 1974, 186 (4159), 143–144. 10.1126/science.186.4159.143. 17744221

[ref11] Bonner W. A.; Kavasmaneck P. R.; Martin F. S.; Flores J. J. Asymmetric adsorption by quartz: A model for the prebiotic origin of optical activity. Origin of LIfe 1975, 6, 367–376. 10.1007/BF01130338. 171608

[ref12] Dutta S.; Gellman A. J. Enantiomer surface chemistry: conglomerate versus racemate formation on surfaces. Chem. Soc. Rev. 2017, 46 (24), 7787–7839. 10.1039/C7CS00555E. 29165467

[ref13] Zaera F. Chiral Modification of Solid Surfaces: A Molecular View. J. Phys. Chem. C 2008, 112 (42), 16196–16203. 10.1021/jp804588v.

[ref14] Baiker A. Chiral catalysis on solids. Curr. Opin. Solid State Mater. Sci. 1998, 3 (1), 86–93. 10.1016/S1359-0286(98)80070-3.

[ref15] Mallat T.; Orglmeister E.; Baiker A. Asymmetric catalysis at chiral metal surfaces. Chem. Rev. 2007, 107 (11), 4863–4890. 10.1021/cr0683663. 17927256

[ref16] Raval R. Chiral expressions at metal surfaces. Curr. Opin. Solid State Mater. Sci. 2003, 7 (1), 67–74. 10.1016/S1359-0286(03)00005-6.

[ref17] Raval R. Chiral expression from molecular assemblies at metal surfaces: insights from surface science techniques. Chem. Soc. Rev. 2009, 38 (3), 707–721. 10.1039/b800411k. 19322464

[ref18] Ernst K. H. Molecular chirality at surfaces. Physica Status Solidi B-Basic Solid State Physics 2012, 249 (11), 2057–2088. 10.1002/pssb.201248188.

[ref19] Gellman A. J.; Ernst K. H. Chiral Autocatalysis and Mirror Symmetry Breaking. Catal. Lett. 2018, 148 (6), 1610–1621. 10.1007/s10562-018-2380-x.

[ref20] Kuzmenko I.; Weissbuch I.; Gurovich E.; Leiserowitz L.; Lahav M. Aspects of spontaneous separation of enantiomers in two- and three-dimensional crystals. Chirality 1998, 10 (5), 415–424. 10.1002/(SICI)1520-636X(1998)10:5<415::AID-CHIR7>3.0.CO;2-4.

[ref21] Jacques J.; Collet A.; Wilen S. Enantiomers, Racemates and Resolutions; Krieger Publishing Company: Malabar, FL, 1991.

[ref22] Marti E. M.; Barlow S. M.; Haq S.; Raval R. Bonding and assembly of the chiral amino acid S-proline on Cu(110): the influence of structural rigidity. Surf. Sci. 2002, 501 (3), 191–202. 10.1016/S0039-6028(01)02026-X.

[ref23] Forster M.; Dyer M. S.; Persson M.; Raval R. Tailoring Homochirality at Surfaces: Going Beyond Molecular Handedness. J. Am. Chem. Soc. 2011, 133 (40), 15992–16000. 10.1021/ja202986s. 21882841

[ref24] Mark A. G.; Forster M.; Raval R. Recognition and Ordering at Surfaces: The Importance of Handedness and Footedness. ChemPhysChem 2011, 12 (8), 1474–1480. 10.1002/cphc.201001034. 21523877

[ref25] Voigt J.; Roy M.; Baljozovic M.; Wackerlin C.; Coquerel Y.; Gingras M.; Ernst K. H. Unbalanced 2D Chiral Crystallization of Pentahelicene Propellers and Their Planarization into Nanographenes. Chem. Eur. J. 2021, 27 (40), 10251–10254. 10.1002/chem.202101223. 34042228 PMC8362048

[ref26] Dutta S.; Yun Y. J.; Widom M.; Gellman A. J. 2D Ising Model for Adsorption-induced Enantiopurification of Racemates. ChemPhysChem 2021, 22 (2), 197–203. 10.1002/cphc.202000881. 33336873

[ref27] Yun Y. J.; Gellman A. J. Adsorption-induced auto-amplification of enantiomeric excess on an achiral surface. Nat. Chem. 2015, 7 (6), 520–525. 10.1038/nchem.2250. 25991532

[ref28] Dutta S.; Gellman A. J. 2D Ising Model for Enantiomer Adsorption on Achiral Surfaces: L- and D-Aspartic Acid on Cu(111). Entropy 2022, 24 (4), 565 10.3390/e24040565. 35455228 PMC9026815

[ref29] Yun Y. J.; Gellman A. J. Competing Forces in Chiral Surface Chemistry: Enantiospecificity versus Enantiomer Aggregation. J. Phys. Chem. C 2016, 120 (48), 27285–27295. 10.1021/acs.jpcc.6b07758.

[ref30] Yun Y. J.; Gellman A. J. Enantioselective Separation on Naturally Chiral Metal Surfaces: d,l-Aspartic Acid on Cu(3,1,17)(R&S) Surfaces. Angew. Chem.-Int. Ed. 2013, 52 (12), 3394–3397. 10.1002/anie.201209025. 23404826

[ref31] McFadden C. F.; Cremer P. S.; Gellman A. J. Adsorption of chiral alcohols on ’’chiral’’ metal surfaces. Langmuir 1996, 12 (10), 2483–2487. 10.1021/la950348l.

[ref32] Mhatre B. S.; Dutta S.; Reinicker A.; Karagoz B.; Gellman A. J. Explosive enantiospecific decomposition of aspartic acid on Cu surfaces. Chem. Commun. 2016, 52 (98), 14125–14128. 10.1039/C6CC06887A. 27868121

[ref33] Karagoz B.; Reinicker A.; Gellman A. J. Kinetics and mechanism of aspartic acid adsorption and its explosive decomposition on Cu(100). Langmuir 2019, 35, 2925–2933. 10.1021/acs.langmuir.8b03482. 30681872

[ref34] Kondratyuk P.; Karagoz B.; Yun Y. J.; Gellman A. J. Initiation of Vacancy-Mediated, Surface Explosion Reactions: Tartaric and Aspartic Acid on Cu Surfaces. J. Phys. Chem. C 2019, 123 (31), 18978–18985. 10.1021/acs.jpcc.9b03895.

[ref35] Yun Y.; Kondratyuk P.; Gellman A. J. Steady-state catalytic decomposition of aspartic acid on Cu(111). Journal of Physical Chemistry - C 2019, 123 (13), 7594–7603. 10.1021/acs.jpcc.8b01923.

[ref36] Yitamben E. N.; Niebergall L.; Rankin R. B.; Iski E. V.; Rosenberg R. A.; Greeley J. P.; Stepanyuk V. S.; Guisinger N. P. Tracking Amino Acids in Chiral Quantum Corrals. J. Phys. Chem. C 2013, 117 (22), 11757–11763. 10.1021/jp400074r.

[ref37] Baldanza S.; Cornish A.; Nicklin R. E. J.; Zheleva Z. V.; Held G. Surface chemistry of alanine on Cu{111}: Adsorption geometry and temperature dependence. Surf. Sci. 2014, 629, 114–122. 10.1016/j.susc.2014.04.016.

[ref38] Merz L.; Ernst K. H. Unification of the matrix notation in molecular surface science. Surf. Sci. 2010, 604 (11–12), 1049–1054. 10.1016/j.susc.2010.03.023.

[ref39] Forster M.; Dyer M. S.; Persson M.; Raval R. Probing Conformers and Adsorption Footprints at the Single-Molecule Level in a Highly Organized Amino Acid Assembly of (S)-Proline on Cu(110). J. Am. Chem. Soc. 2009, 131 (29), 10173–10181. 10.1021/ja9020364. 19580280

[ref40] Eralp T.; Shavorskiy A.; Zheleva Z. V.; Held G.; Kalashnyk N.; Ning Y. X.; Linderoth T. R. Global and Local Expression of Chirality in Serine on the Cu{110} Surface. Langmuir 2010, 26 (24), 18841–18851. 10.1021/la1036772. 21090821

[ref41] Cundy K. C.; Crooks P. A. Unexpected Phenomenon in the High-Performance Liquid-Chromatographic Analysis of Racemic ^14^C-labelled Nicotine - Separation of Enantiomers in a Totally Achiral System. J. Chromatogr. 1983, 281 (DEC), 17–33. 10.1016/S0021-9673(01)87863-8.

[ref42] Soloshonok V. A.; Roussel C.; Kitagawa O.; Sorochinsky A. E. Self-disproportionation of enantiomers via achiral chromatography: a warning and an extra dimension in optical purifications. Chem. Soc. Rev. 2012, 41 (11), 4180–4188. 10.1039/c2cs35006h. 22517405

