# Supplementary material for: Molecular Origins of Chiral Amplification on an Achiral Surface: 2D Monolayers of Aspartic Acid on Cu(111)
Source: ACS Nano. 2023 Mar 6;17(6):5799–807. doi: 10.1021/acsnano.2c12312 (PMC10062026; doi:10.1021/acsnano.2c12312)
Supplement: Supplementary file 1 — nn2c12312_si_001.pdf [file nn2c12312_si_001.pdf]

Supporting Information

Molecular origins of chiral amplification on an achiral surface: 2D monolayers of aspartic acid on Cu(111)

Laura A. Cramer[1], Amanda Larson[1], Avery S. Daniels[1],
E. Charles H. Sykes[1], Andrew J. Gellman[2,3,*]

[1]Department of Chemistry, Tufts University, Medford, MA 02155-5813
[2]Department of Chemical Eng.,
[3]W.E. Scott Institute for Energy Innovation,
Carnegie Mellon University, Pittsburgh, PA 15213

SI1. Unit cell assignment for Asp/Cu(111)

The matrix notation assignments of the unit cells of the $T1_D$, $T1_L$, $T2_D$, $T2_L$, HC_D and HC_L Asp/Cu(111) overlayers have been determined by identifying the unit cell vectors from the images of the overlayer (Figures 2 and 3). These vectors were then superimposed onto a scaled image of the Cu(111) surface (Figure 3c) and rotated to identify the orientation that is commensurate with the Cu(111) substrate. They were then rotated by 0˚, 120˚ or 240˚ to identify the Master matrix as determine by the convention of Ernst.[38]

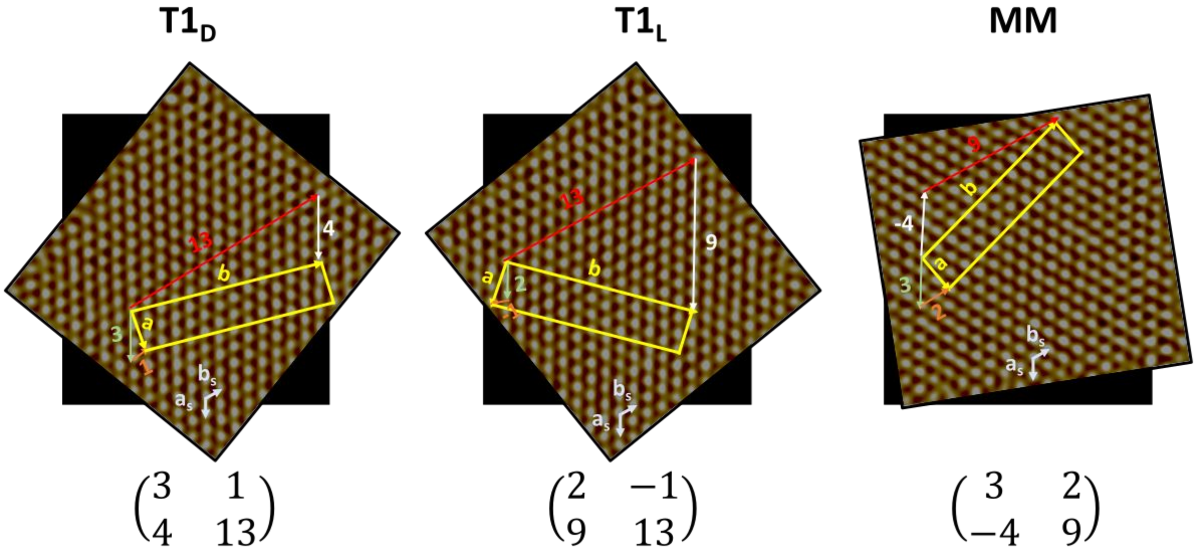

Figure SI1. Unit cell vectors (yellow) for the $T1_D$, $T1_L$, and master matrix (MM) overlayers superimposed on the STM image of the Cu(111) surface.

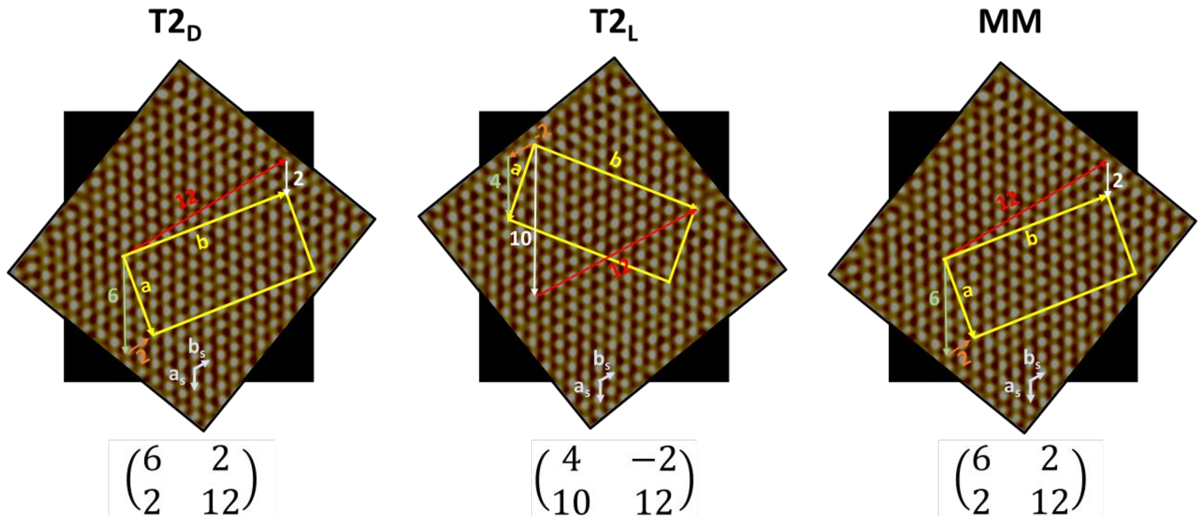

Figure SI2. Unit cell vectors (yellow) for the $T2_D$, $T2_L$, and master matrix (MM) overlayers superimposed on the STM image of the Cu(111) surface.

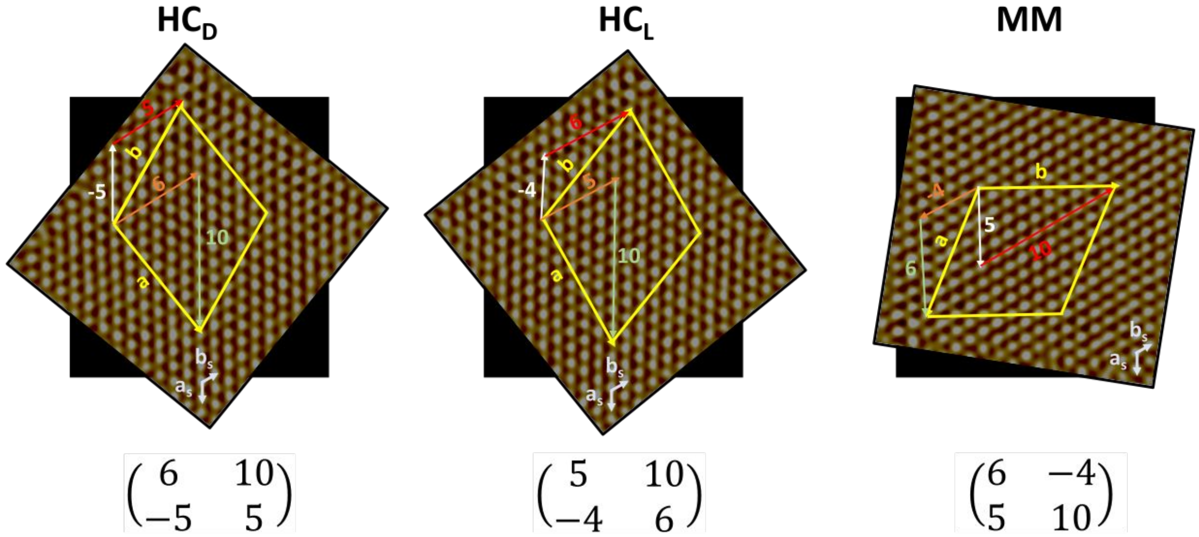

Figure SI3. Unit cell vectors (yellow) for the HC_D, HC_L, and master matrix (MM) overlayers superimposed on the STM image of the Cu(111) surface.

SI2. Enantiomer distribution in unit cells of Asp/Cu(111)

Our STM resolution does not yield submolecular structure such as molecular orientation or chirality. However, based on the locations of features that are observed in the images we have made a hypothetical proposal for the distribution of enantiomers in the observe overlayer structures. These are consistent with our estimates of the proposed enantiomer compositions.

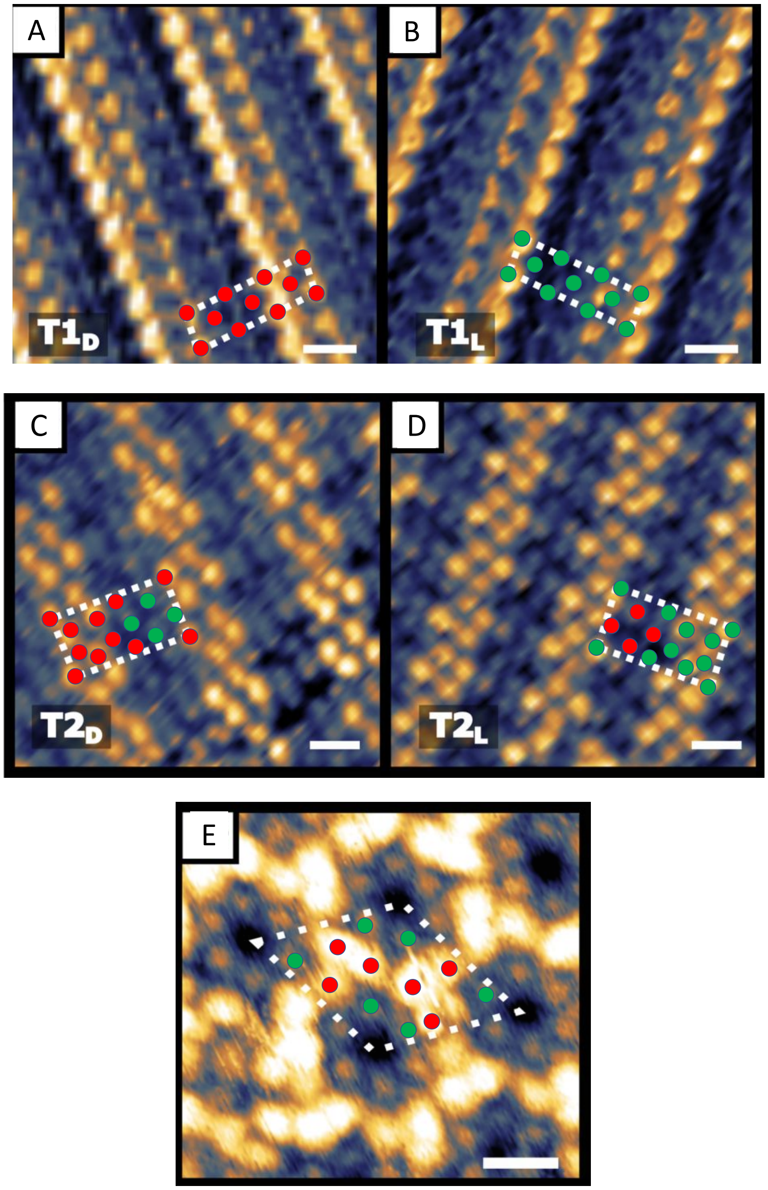

Figure SI4. Representations of possible locations for adsorption of D-Asp (red circles) and L-Asp (green circles) enantiomers in the three overlayer structures observed on Cu(111): A) $T1_D$, B) $T1_L$, C) $T2_D$, D) $T2_L$, E) HC. The surface enantiomeric excesses are: $ee_s =$ A) 1, B) -1, C) 0.33, D) -0.33, E) 0.
